# Are clinicians overdiagnosing strep throat and overprescribing antibiotics?

**DOI:** 10.3389/fped.2023.1248903

**Published:** 2023-11-03

**Authors:** Kennedy A. Sabharwal, Michael W. Simon

**Affiliations:** ^1^ Department of Medicine, University of Kentucky College of Medicine, Lexington, KY, United States; ^2^ Department of Pediatrics, University of Kentucky, Lexington, KY, United States

**Keywords:** strep throat, streptococcus, Group A streptococci, children, Strep A

## Introduction

Clinicians may be over diagnosing Group A beta hemolytic streptococci (GABHS)/*Streptococcus pyogenes* illness due to current testing methods. According to the name-branded OSOM Strep A Test and QuickVue Strep A Test, these forms of tests are intended for qualitative detection of the GABHS antigen from throat swabs. Although GABHS is the most common bacterium, causing 15%–30% of acute sore throat or acute attacks of recurrent tonsilitis, swab cultures taken from the tonsillar surface may only reflect colonization and may not represent the actual bacterial pathogen of the internal tonsil core that is causing the acute infection (1).

According to an observational prospective study with 77 children <15 years old with sore throat and 34 asymptomatic children, many other bacteria and viruses may present with similar symptoms as GABHS (2). These symptoms include, but are not limited to, days with sore throat prior to visit with clinician, cough, coryza, tonsillar coating, and swollen tonsils. This study investigated the broad spectrum of bacteria and viruses that cause pediatric pharyngotonsillitis, while also focusing on the fact that GABHS was found in 12% of their asymptomatic children. This shows there is a real threat of overdiagnosing Group A Streptococcal illness in children with sore throat and the unnecessary use of antibiotics contributing to antibiotic resistance.

As mentioned above, the current gold standard testing for Group A streptococci uses throat swabs which only qualitatively detect the presence of the bacteria. In the past, healthcare providers would test for GABHS by using throat culture on blood agar plates. A rule of thumb was if there were fewer than 10 colonies recovered from the culture, the physician would classify the patient as a carrier of the bacteria. However, a larger number of colonies recovered would be consistent with and represent an acute infection with Group A streptococcal bacteria.

When comparing the current testing method of a rapid strep test to the QuickVue 5% sheep blood agar plate test stored at room temperature, results are as follows:

If there are 10 or fewer GABHS colonies confirmed on the blood agar plate, there is a less than 50% chance of having a positive rapid strep test.

If there are between 11 and 50 GABHS colonies confirmed on the blood agar plate, there is an 80% chance of having a positive rapid strep test.

If there are 50–100 GABHS colonies confirmed on the blood agar plate, there is a 95% chance of having a positive rapid strep test (3).

In addition, the QuickVue blood agar plate strep test performed culture confirmation of Group A *Streptococcus* which was 100% sensitive. No cross-reactivity with other organisms was seen. The QuickVue test also showed to have a 92% sensitivity and a 98% specificity (3). Positive results seen in a rapid strep test are also dependent on the quality of sample that is collected during testing.

Since the current gold standard testing only shows the presence of the bacteria and not a quantity of bacteria colonies that are grown, children who are asymptomatic carriers of GABHS, but ill with other acute infections could be falsely diagnosed with GABHS and unnecessarily prescribed antibiotics. The recommended antibiotics for GABHS infection are penicillin and amoxicillin. Overprescribing and resistance to antibiotics can lead to larger issues as seen in recurrent tonsillitis. In recurrent tonsillitis, the goal of treatment is to eradicate the bacteria that cause the infection; however, antibiotic therapy when not required or the wrong choice against the pathogen in deep tissue may lead to the continuation of the infection, causing the patient to have chronic tonsillitis. Specifically, chronic colonization of the tonsils with *Haemophilus Influenzae* has been assessed due to its association with adenoid hyperplasia (4).

Many studies have compared tonsillar surface and core culture isolates to observe the difference between bacteria that may be causing acute tonsillitis. It had previously been claimed that the results of throat culture swabs may not always show the true pathogen that is causing disease (5). A newer study with 116 participants investigated the differences in cultures of the tonsillar surface and deep tonsillar tissue cultures. This study found that swab cultures may not always reveal the true pathogen causing acute infection, and it would be beneficial to prescribe additional antibiotics against *H influenzae* (e.g., broad-spectrum cephalosporins), as well as the other infecting organism when treating acute sore throat (6). It is important to point out that the results of this study varied depending on vaccination status, specifically with the pneumococcal and Hib vaccines. Thus, if many studies are beginning to prove that surface throat cultures do not match deep tissue tonsillar cultures, resulting in incorrect and/or overprescription of antibiotics. This may be a leading factor for chronic tonsillitis. This should be factored when considering diagnosis of GABHS from surface samples. With the dependency and reliance healthcare providers have upon the rapid strep test, clinicians may be overdiagnosing and overtreating individuals who truly do not have an acute strep throat infection.

This brings us to the question of how clinicians ought to proceed in diagnosing and treating children with an acute sore throat. Clinicians cannot rely solely on the results of a rapid strep test. Clinicians must base the diagnosis upon characteristic throat and palatal findings coupled with a rapid strep test to diagnose strep throat and prescribe an appropriate antibiotic more confidently. Clinicians should continue to utilize the rapid strep test to confirm their clinical findings and history for strep throat. Combining both clinical findings and antigen test results increases both the sensitivity and specificity of the strep throat diagnosis. To increase the accuracy (including sensitivity and specificity), anyone performing a rapid strep test must be appropriately trained in their proper sample collection.

Other research has begun to explore the efficacy of various testing methods for GABHS. A study published in the *Journal of Clinical Microbiology* showed a greater sensitivity, but decreased specificity in detection of GABHS by using a chromogenic detection module software called PhenoMATRIX to observe agar plates compared to manual reading of agar plates (7). Another recent study compared the sensitivity and specificity of two current rapid strep tests and assessed the overall performance of rapid strep tests in general. This study mentioned that rapid strep tests with higher diagnostic specificity are more important to use than those with higher diagnostic sensitivity to minimize the inappropriate use of antibiotics in patients who are only GABHS carriers (8). However, we cannot have an overreliance of diagnostic test results. They must be coupled with consistent history and physical findings.

In summary, overprescription of antibiotics in the case of acute sore throat is an issue that must be considered widely across the medical field. Moving forward, it may be beneficial to use rapid strep tests with the highest diagnostic sensitivity so as not to miss false negatives and develop a new strep test that may quantitatively assess the amount of GABHS bacteria present. The overall goal is to appropriately use antibiotics in patients by ensuring that the diagnostic methods, when coupled with physical findings and history, are correctly identifying strep carriers, and not missing those with strep illness. If there is a high index of clinical suspicion but with a negative rapid strep test the provider is uncertain, treatment may be delayed and give another 24 h for the diagnosis to be more certain.
